# Ofatumumab for B cell depletion therapy in ANCA-associated vasculitis: a single-centre case series

**DOI:** 10.1093/rheumatology/kew199

**Published:** 2016-04-19

**Authors:** Stephen P. McAdoo, Rachna Bedi, Ruth Tarzi, Megan Griffith, Charles D. Pusey, Thomas D. Cairns

**Affiliations:** ^1^Vasculitis Centre, Imperial College Healthcare NHS Trust; ^2^Renal and Vascular Inflammation Section, Department of Medicine, Imperial College London, Hammersmith Hospital, London, UK

**Keywords:** ANCA, vasculitis, B cells, biologic therapies, microscopic polyangiitis, granulomatosis with polyangiitis

## Abstract

**Objectives.** B cell depletion is an effective treatment strategy in ANCA-associated vasculitis (AAV). Ofatumumab is a fully humanized anti-CD20 mAb that has shown efficacy in the treatment of haematological malignancy and RA. The use of ofatumumab in the treatment of AAV has not previously been reported.

**Methods.** This study was based on a case series of eight patients who received ofatumumab, in conjunction with low-dose CYC and oral steroids, in the treatment of AAV.

**Results.** Eight patients received ofatumumab: seven for remission induction in active disease (three relapsing; four with new disease) and one for remission maintenance. B cell depletion was achieved in all patients by 1 month, and was sustained for at least 6 months. All patients with active disease achieved clinical remission (BVAS of zero, or BVAS ⩽5 if all scores due to persistent urinary abnormalities in the presence of stable or improving renal function) by 3 months. This was associated with a rapid fall in ANCA titres, reduced inflammatory responses and improvements in renal function. At 12 months, three patients had repopulated B cells associated with the recurrence of circulating ANCAs, although no patients experienced major clinical relapse in the first 24 months. No unexpected side effects were observed.

**Conclusion.** Treatment with ofatumumab resulted in similar serological and clinical responses to those seen in previous cohorts treated at our centre with a comparable CS, CYC and rituximab-based regimen. Ofatumumab should be considered an alternative B cell depleting agent in patients who are intolerant of, or unresponsive to, rituximab.

Rheumatology key message

- Ofatumumab is a potential alternative to rituximab for B cell depletion therapy in ANCA-associated vasculitis.

## Introduction

Targeted B cell depletion has emerged as an effective treatment strategy in ANCA-associated vasculitis (AAV), both as remission-induction and remission-maintenance therapy [1–3]. All reported series and trials using this approach have employed rituximab, a murine chimeric mAb directed against the transmembrane calcium channel CD20, which results in predominantly antibody-dependent cellular cytotoxicity-mediated depletion of circulating B lymphocytes expressing this molecule.

Ofatumumab is a fully humanized mAb directed against a distinct extracellular epitope of CD20, which has slower dissociation kinetics compared with rituximab, and which has been shown to be a more potent activator of complement-dependent cytotoxicity *in vitro* [4]. It is licensed for use in haematological malignancies, where it has shown biologic activity in rituximab-resistant disease [5, 6]. Notably, ofatumumab has also shown efficacy in rituximab-resistant cases of paediatric nephrotic syndrome [7], and has demonstrated biological activity in RA [8–10].

Here, we report our preliminary experience using ofatumumab for the treatment of AAV. This was based on experience of ofatumumab use in patients with LN who had demonstrated anaphylactic reactions to rituximab, and was initially used in a patient with AAV who had similarly demonstrated an anaphylactic reaction to rituximab. This approach was subsequently extended to include rituximab-naïve patients, based on our positive initial experience. Our treatment regimen using ofatumumab was based on our previously published protocol for using rituximab (in conjunction with low-dose pulsed i.v. CYC and a reduced steroid dose) [11]. This regimen, which has been standard of care for AAV with renal involvement at our centre since 2006, is associated with low cumulative exposure to CYC and steroids, and prolonged disease-free remission. In the present study, ofatumumab was substituted in for rituximab at the initiation of remission-induction therapy, and we report 2-year outcomes.

## Methods

This study is based on a case series of eight patients treated between November 2012 and July 2013 at our centre, who received ofatumumab as a component of their treatment regimen for AAV. Ofatumumab was initially used at our centre on compassionate grounds for patients who were intolerant to rituximab due to anaphylaxis, but for whom anti-CD20 treatment was deemed appropriate (both in LN and AAV; the lupus cohort will be reported separately). Following our positive initial experience in these patients, rituximab-naive patients with AAV were also treated. Ofatumumab use was off-label, based on evidence supporting the use of anti-CD20 therapy in AAV at that time, and predated the licensing of rituximab for this indication in 2013 (when use of the latter agent was similarly off-label). Use of ofatumumab was guided by expert opinion at our centre and approved by the Glomerulonephritis Protocol and Research Group at Imperial College Healthcare National Health Service Trust.

The treatment protocol was based on our previously reported regimen using low-dose pulsed i.v. CYC and steroids, in conjunction with anti-CD20 treatment [11], and this has been standard of care at our centre since 2006. Herein, ofatumumab 2 × 700 mg was substituted in for rituximab 2 × 1 g administered at days 0 and 14. The additional components of the protocol included i.v. CYC 10 mg/kg administered at days 0 and 14 (maximum 750 mg each) and then every 14 days for a further four doses (maximum 500 mg each). Oral prednisolone 1 mg/kg was started at day 0 (maximum 60 mg) and reduced sequentially to achieve a dose of 10 mg by week 13. Further steroid weaning was at the discretion of the treating physician. Where patients were treated for relapsing disease or for remission maintenance, or had received pulsed i.v. methylprednisolone before referral to our centre, modified doses of either steroids or CYC may have been used. Therapeutic plasma exchange was offered for patients who presented with dialysis-dependent renal failure. In these cases, administration of the first dose of ofatumumab was delayed until completion of plasma exchange. Maintenance therapy commenced at 3 months, after completion of cytotoxic therapy, or earlier in those who received modified doses of CYC. First-line maintenance was with AZA; MMF was used in those who were intolerant. Patients received prophylactic co-trimoxazole for 3 months, proton pump inhibitors and bone protection with calcium/D3. Patients from high-risk groups were also given prophylaxis against tuberculosis with isoniazid and pyridoxine.

Patients were regularly assessed using both clinical and laboratory measures. Disease activity was scored using version 3 of the BVAS [12]. Laboratory assessments included monitoring of the B cell count, ANCA titre, serum CRP and renal function as determined by serum creatinine measurements.

Informed consent was provided prior to initiation of therapy in all cases. In accordance with the UK National Health Service Research Ethics Committee guidelines, ethics approval was not required for this report, because all treatment decisions were made prior to this evaluation, and were part of a formal change in our standard-care regimen

## Results

Individual patient characteristics and details of the treatments are summarized in Table 1. The demographic features of this cohort are in keeping with that of the population of patients with vasculitis seen at our centre. The cohort included a mixture of AAV disease phenotypes, including granulomatosis with polyangiitis, microscopic polyangiitis and eosinophilic granulomatosis with polyangiitis (EGPA). Five of the six patients with renal involvement had confirmatory renal biopsies demonstrating necrotizing or crescentic glomerulonephritis. Two patients were ANCA-negative; of these, one had a renal biopsy confirming necrotizing glomerulonephritis consistent with small-vessel vasculitis (case 5). The second ANCA-negative patient had an established diagnosis of EGPA based on prior presentation with adult-onset asthma, sinusitis, biopsy-proven granulomatous panniculitis and peripheral blood eosinophilia (case 3). Two patients presented with dialysis-dependent renal failure and received plasma exchange in addition to medical therapy with anti-CD20, CYC and CSs. Three patients were treated for relapsing disease, one for maintenance of remission in EGPA (in response to rising ANCA titres and eosinophilia without clinical manifestations), and four patients for new disease presentations. Of the two patients who had previously received anti-CD20, one had demonstrated an anaphylactic infusion reaction to rituximab (case 1) and on this basis was treated with ofatumumab.

**Table 1:** Clinical features and treatment regimens for individual patients receiving ofatumumab

Characteristics	Case 1	Case 2	Case 3	Case 4	Case 5	Case 6	Case 7	Case 8
Age, years	25	69	53	51	77	78	21	43
Gender	F	M	F	F	M	M	M	M
Ethnicity	Caucasian	Caucasian	North African	Caucasian	South Asian	Caucasian	South Asian	Caucasian
Clinical diagnosis	GPA	MPA	EGPA	EGPA	MPA	GPA	GPA	MPA
ANCA IIF	cANCA ++	pANCA+	Negative	cANCA ++	Negative	cANCA ++	cANCA ++	pANCA ++
ANCA ELISA specificity	PR3	Negative	Negative	PR3	Negative	PR3	PR3	MPO
ANCA ELISA titre (NR <25)	252	Negative	Negative	139	Negative	573	962	215
Treatment indication	Relapse	Relapse	Relapse	Remission maintenance	New disease	New disease	New disease	New disease
For relapsing/remission maintenance patients								
Duration of AAV, years	10	47	5	15	—	—	—	—
Previous relapses	3	1	5	3	—	—	—	—
Major	2	0	0	2				
Minor	1	1	5	1				
Previous cumulative CYC, g	3	0	0	10	—	—	—	—
Previous cumulative RTX, g	2	0	0	2	—	—	—	—
Other previous treatments	CS	CS only			—	—	—	—
	AZA		CS	CS				
	MMF		AZA	AZA				
	MTX			MMF				
	HCQ							
Time since last RTX (BCC at this presentation, cells/µl)	62 months (175)	—	—	15 months (38)	—	—	—	—
Organ Involvement at this Presentation	Renal			Eosinophilia, rising ANCA titre	Renal	Renal	Renal	
	ENT	Renal	ENT		Lung	ENT	ENT	Renal
	Skin	Skin	Lung		Neuro	Lung	Lung	ENT
	MSK	MSK	Neuro		GI	Hepatic	MSK	
BVAS at this presentation	19	16	10	0	26	24	23	18
Treatment								
Ofatumumab dose (i.v.), mg	2 × 700	2 × 700	2 × 700	2 × 700	2 × 700	2 × 700	2 × 700	2 × 700
CYC dose (i.v.), g	3.0	3.2	—	—	1.0	3.1	3.2	3.6
Methylprednisolone dose (i.v.), mg	1000	—	—	—	—	500	—	—
Initial prednisolone, dose (p.o.), mg	40	60	30	5	60	60	60	60
PEX, n	—	—	—	—	—	7	—	7
Maintenance	MMF	MMF	AZA	MMF	MMF	AZA	AZA	AZA

AAV: ANCA-associated vasculitis; ANCA: antineutrophil cytoplasm antibody; BCC: B cell count; BVAS: Birmingham vasculitis activity score; EGPA: eosinophilic granulomatosis with polyangiitis; ELISA: enzyme-linked immunosorbent assay; GI: gastrointestinal; GPA: granulomatosis with polyangiitis; IIF: indirect immunofluorescence; MPA: microscopic polyangiitis; MSK: musculoskeletal; NR: normal range; PEX: plasma exchange; RTX: rituximab.

**Fig. 1:**
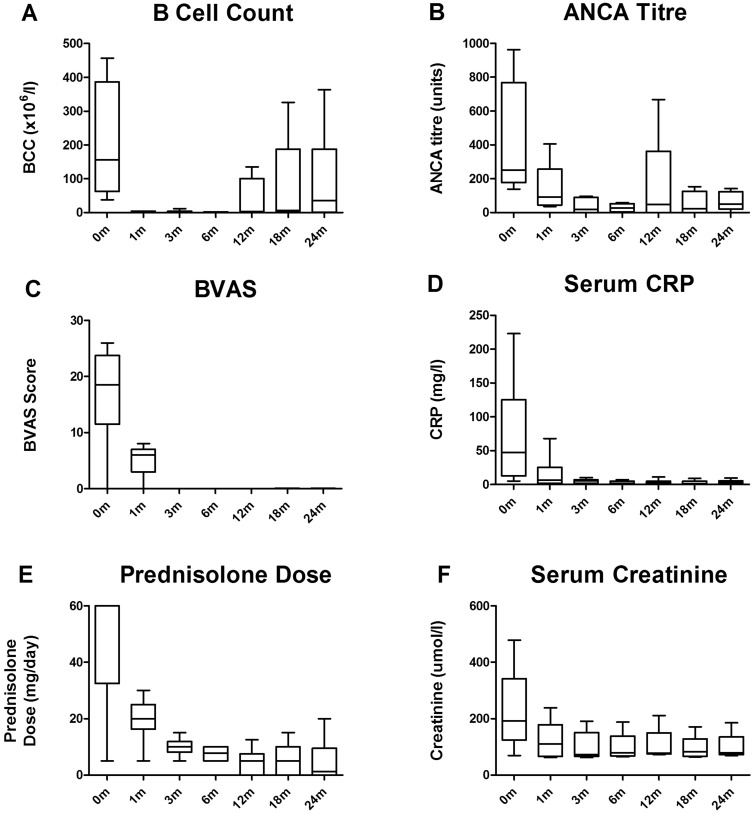
Serological and clinical responses at 0, 1, 3, 6, 12, 18 and 24 months following ofatumumab treatment Box-and-whisker plots describe the median, interquartile range and minimum and maximum values. One patient who presented with dialysis-dependent renal failure and 50% interstitial fibrosis and tubular atrophy on renal biopsy, who did not recover independent renal function, has been excluded from Fig. 1F. Similarly, this case has been censored from all other figures following renal transplantation at 21 months. BCC: B cell count.

All patients experienced B cell depletion by 1 month, as defined by an absolute B cell count of <5 cells/μl (Fig. 1A). B cell depletion was sustained until at least 6 months in all patients. This was associated with a rapid reduction in ANCA titres (Fig. 1B). All patients with active disease achieved clinical remission by 3 months, as defined by BVAS of zero or BVAS ⩽5 if all scores were due to persistent haematuria or proteinuria in the presence of stable or improving renal function as measured by serum creatinine (Fig. 1C). This was associated with a reduced acute phase response (Fig. 1D) and the ability to taper CS treatment (Fig. 1E). The median total dose of CYC administered was 3.05 g. In those patients with renal involvement (n = 6), there was improvement in renal function in five cases (Fig. 1F). One patient who presented with dialysis-dependent renal failure and 50% interstitial fibrosis and tubular atrophy on renal biopsy, did not recover independent renal function (case 8). His other disease manifestations did, however, resolve (BVAS 0 at 3 months), and he remained in stable remission, allowing successful renal transplantation at 21 months. This was with alemutuzumab-based induction and tacrolimus monotherapy for immunosuppression [13]. Accordingly, he remains lymphocyte deplete, with no clinical features of vasculitis and no evidence of disease recurrence on allograft surveillance biopsy.

At 12 months, three patients had repopulated B cells (absolute count >20 cells/μl), in all cases associated with recurrence of circulating ANCA (cases 2, 6 and 7), although no patients experienced clinical relapse in the first year. At 24 months, a further patient reconstituted B cells with weakly positive ANCA, though without clinical features of relapse (case 1). In those patients who reconstituted B cells over the 2-year follow-up period, the median time to reconstitution was 11.8 months. After 2 years follow-up, one ANCA-negative patient with EGPA (case 3) experienced two minor relapses, in the absence of B cell reconstitution, at 15 and 21 months, which were treated with oral CSs only.

We did not detect any unexpected side effects: one patient experienced a minor infusion reaction to ofatumumab; three patients had infectious complications (in total there were three urinary tract infections, one upper respiratory tract infection and one lower respiratory tract infection); one patient had a transient episode of neutropenia not associated with infection. There were no atypical or opportunistic infections, and we did not detect any episodes of hypogammaglobulinaemia or hepatitis reactivation.

### Conclusion

This is the first reported use of ofatumumab in AAV, and our preliminary data suggest that this agent results in similar serological and clinical responses to those of previous cohorts treated with a comparable CS, CYC and rituximab-based regimen at our centre [11], with complete B-cell depletion within 1 month, sustained in half of the patients at 24 months, and associated prolonged relapse-free survival.

Our experience suggests that ofatumumab warrants further investigation as an alternative B cell–depleting agent in AAV. This may be of particular use in patients who are intolerant of rituximab due to infusion-related reactions. Indeed, there is a previous case report of successful ofatumumab use in a patient with SLE who developed anaphylaxis following rituximab infusion [14]. Notably, human anti-chimeric antibodies (HACA) have been implicated in the generation of infusion reactions following repeated rituximab exposure (with reported HACA frequencies of up to 11% in some cohorts [15]), though their clinical significance is not fully understood. As a fully humanized antibody, ofatumumab may avoid immunogenic anti-drug reactions [9], which may have additional benefits both for tolerability and efficacy after repeated exposure. Future studies might also explore potential cost benefits of alternative B cell–depleting agents; the cost per course in the UK of using our protocols equates to £2548 (2 × 700 mg ofatumumab) *vs* £3492 (2 × 1 g rituximab).

Our report has obvious limitations; the cases are small in number and heterogeneous, and the series is uncontrolled. Several patients received CYC in addition to ofatumumab, which may have affected both B cell survival and clinical response, though it is notable that complete B cell depletion was observed in both patients who did not receive concurrent CYC. In addition, the median dose of CYC administered (3.05 g) was significantly lower than that reported in published studies using CYC alone as remission-induction therapy (e.g. 8.2 and 15.9 g in the pulsed-i.v. and daily-oral groups, respectively, in the CYCLOPS study [16]). In addition, the inclusion of low-dose CYC treatment for patients with severe renal disease is consistent with the use of rituximab in the RITUXVAS study [2]. Notably, the rapidity, depth and duration of B cell depletion that we observed in our cohort is greater than that seen in the published studies using CYC alone (e.g. <10% of patients in the control limbs of the RITUXVAS and RAVE studies achieved B cell depletion within the first month [17, 18]). Collectively, these observations suggest that a significant proportion of the B cell–depleting and therapeutic effect seen in our cohort is due to the activity of ofatumumab. We acknowledge, however, that the synergistic effect of co-administration of anti-CD20 with low-dose CYC is not fully understood and requires further investigation.

Finally, we have not examined the utility of ofatumumab in rituximab-resistant disease, for which there is evidence of benefit in the treatment of paediatric nephrotic syndrome and haematological malignancy, potentially due to differences in drug epitope specificity, pharmacokinetics or the potential to avoid induction of HACA. Larger studies, including randomized controlled trials, are clearly needed to address these questions and to more precisely define the role of this and other emerging anti-B cell therapies in AAV. Pending these studies, this series provides a potential dosing regimen and preliminary evidence that ofatumumab may be a useful alternative agent in the treatment of AAV.

## References

[1] Stone JH Merkel PA Spiera R Rituximab versus cyclophosphamide for ANCA-associated vasculitis. New Engl J Med. 2010;363:221–32. 20647199 10.1056/NEJMoa0909905 PMC3137658

[2] Jones RB Tervaert JW Hauser T Rituximab versus cyclophosphamide in ANCA-associated renal vasculitis. New Engl J Med 2010;363:211–20. 20647198 10.1056/NEJMoa0909169

[3] Guillevin L Pagnoux C Karras A Rituximab versus azathioprine for maintenance in ANCA-associated vasculitis. New Engl J Med 2014;371:1771–80. 25372085 10.1056/NEJMoa1404231

[4] Zhang B. Ofatumumab. mAbs 2009;1:326–31. 20068404 10.4161/mabs.1.4.8895 PMC2726602

[5] Czuczman MS Fayad L Delwail V Ofatumumab monotherapy in rituximab-refractory follicular lymphoma: results from a multicenter study. Blood 2012;119:3698–704. 22389254 10.1182/blood-2011-09-378323

[6] Matasar MJ Czuczman MS Rodriguez MA Ofatumumab in combination with ICE or DHAP chemotherapy in relapsed or refractory intermediate grade B-cell lymphoma. Blood 2013;122:499–506. 23692856 10.1182/blood-2012-12-472027 PMC3724189

[7] Basu B. Ofatumumab for rituximab-resistant nephrotic syndrome. New Engl J Med 2014;370:1268–70. 24670185 10.1056/NEJMc1308488

[8] Østergaard M Baslund B Rigby W Ofatumumab, a human anti-CD20 monoclonal antibody, for treatment of rheumatoid arthritis with an inadequate response to one or more disease-modifying antirheumatic drugs: results of a randomized, double-blind, placebo-controlled, phase I/II study. Arthritis Rheum 2010;62:2227–38. 20506254 10.1002/art.27524

[9] Taylor PC Quattrocchi E Mallett S Ofatumumab, a fully human anti-CD20 monoclonal antibody, in biological-naive, rheumatoid arthritis patients with an inadequate response to methotrexate: a randomised, double-blind, placebo-controlled clinical trial. Ann Rheum Dis 2011;70:2119–25. 21859685 10.1136/ard.2011.151522 PMC3212699

[10] Kurrasch R Brown JC Chu M Subcutaneously administered ofatumumab in rheumatoid arthritis: a phase I/II study of safety, tolerability, pharmacokinetics, and pharmacodynamics. J Rheumatol 2013;40:1089–96. 23729801 10.3899/jrheum.121118

[11] Mansfield N Hamour S Habib AM Prolonged disease-free remission following rituximab and low-dose cyclophosphamide therapy for renal ANCA-associated vasculitis. Nephrol Dial Transplant 2011;26:3280–6. 21414973 10.1093/ndt/gfr127

[12] Mukhtyar C Lee R Brown D Modification and validation of the Birmingham Vasculitis Activity Score (version 3). Ann Rheum Dis 2009;68:1827–32. 19054820 10.1136/ard.2008.101279

[13] Chan K Taube D Roufosse C Kidney transplantation with minimized maintenance: alemtuzumab induction with tacrolimus monotherapy—an open label, randomized trial. Transplantation 2011;92:774–80. 21836540 10.1097/TP.0b013e31822ca7ca

[14] Thornton CC Ambrose N Ioannou Y. Ofatumumab: a novel treatment for severe systemic lupus erythematosus. Rheumatology 2015;54:559–60. 25539826 10.1093/rheumatology/keu475

[15] van Vollenhoven RF Emery P Bingham CO III Longterm safety of patients receiving rituximab in rheumatoid arthritis clinical trials. J Rheumatol 2010;37:558–67. 20110520 10.3899/jrheum.090856

[16] de Groot K Harper L Jayne DR Pulse versus daily oral cyclophosphamide for induction of remission in antineutrophil cytoplasmic antibody-associated vasculitis: a randomized trial. Ann Int Med 2009;150:670–80. 19451574 10.7326/0003-4819-150-10-200905190-00004

[17] Jones RB Furuta S Tervaert JW Rituximab versus cyclophosphamide in ANCA-associated renal vasculitis: 2-year results of a randomised trial. Ann Rheum Dis 2015;74:1178–82. 25739829 10.1136/annrheumdis-2014-206404

[18] Specks U Merkel PA Seo P Efficacy of remission-induction regimens for ANCA-associated vasculitis. New Engl J Med 2013;369:417–27. 23902481 10.1056/NEJMoa1213277 PMC5953195

